# Supplementary material for: Implementing artificial intelligence in Canadian primary care: Barriers and strategies identified through a national deliberative dialogue
Source: PLoS One. 2023 Feb 27;18(2):e0281733. doi: 10.1371/journal.pone.0281733 (PMC9970060; doi:10.1371/journal.pone.0281733)
Supplement: S1 Table — (DOCX) [file pone.0281733.s002.docx]

**S1 Table. Study participants**

*Also presented in work by Kiran et al. (19), currently under review with the* Journal of the American Board of Family Medicine.

|  | **Patients** | **Providers** |
| --- | --- | --- |
| ***Province***n (%) |  |  |
| British Columbia | 1 (4.5) | 1 (4.8) |
| Alberta | 4 (18) | 2 (9.5) |
| Manitoba | - | 5 (24) |
| New Brunswick | 1 (4.5) | - |
| Nova Scotia | 3 (14) | 1 (4.8)**^†^** |
| Ontario | 12 (55) | 11 (52) |
| Quebec | 1 (4.5) | 1 (4.8) |
| ***Age in years***Range, Mean (SD) | 23-73, 40 (16) | 28-64, 42 (8.7) |
| ***Gender***n (%) |  |  |
| Female^††^ | 12 (55) | 9 (43) |
| Male | 9 (41) | 12 (57) |
| Non-binary | 1 (5) | - |
| ***Race or ethnicity***n (%) |  |  |
| Black | 2 (9) | 1 (4.8) |
| East/Southeast Asian | 1 (5) | 2 (9.5) |
| South Asian | 6 (27) | 5 (24) |
| White | 13 (59) | 10 (48) |
| Mixed | - | 3 (14) |
| ***Self-rated AI knowledge***^†††^ Mean (SD) | 2.7 (1.1) | 3.3 (1.2) |
| ***Provider type*** |  |  |
| Chiropractor | - | 1 (4.8) |
| Clerical staff | - | 1 (4.8) |
| Family physician | - | 14 (67) |
| Family medicine resident | - | 2 (9.5) |
| Nurse practitioner | - | 2 (9.5) |
| Social worker | - | 1 (4.8) |
| ***Years in practice*** Mean (SD)^‡^ | - | 12 (10) |
| ***Practice size***^‡‡^ |  |  |
| < 250 patients | - | 6 (30) |
| 250-750 patients | - | 6 (30) |
| 750-1250 patients | - | 5 (25) |
| ***FTE clinical hours per week*** Mean (SD)^‡‡‡^ | - | 0.67 (0.30) |

^†^Canadian province in which Denmark-licensed family physician studied health information technology in primary care settings; ^††^ including one trans woman; ^†††^ Participants rated their knowledge of AI on a five-point Likert scale (1 was “Not knowledgeable at all”, 5 was “extremely knowledgeable”); ^‡^Calculation includes years in residency; ^‡‡^Excluding residents, clerical participant; ^‡‡‡^Full-time equivalent, calculation excludes clerical participant

Due to rounding, some totals may not perfectly sum to 100.
