# Supplementary material for: Implementing artificial intelligence in Canadian primary care: Barriers and strategies identified through a national deliberative dialogue
Source: PLoS One. 2023 Feb 27;18(2):e0281733. doi: 10.1371/journal.pone.0281733 (PMC9970060; doi:10.1371/journal.pone.0281733)
Supplement: S2 Table — (PDF) [file pone.0281733.s003.pdf]

S2: Tables – Iterative Coding Process

1. Use Case Chart: Use cases were used to develop initial perceived barriers and strategies and main takeaways were summarized

<table>
<tr><th>Use Case Description</th><th>Perceived Barriers</th><th>Facilitative Strategies</th><th>Summary/ Main Takeaways of Use Case</th></tr>
<tr><td rowspan="7">Use Case #1 (Risk Stratification Tool)

A large primary care organization that serves 36,000 people is using a new AI tool. It processes the usual health data found in EMRs and combines it with social data that the organization has collected on patients, like their housing, income and employment status, and their language preference. Clinic leadership has asked physicians to spend dedicated time each shift focusing on their top 10 patients who are at risk of getting sick.</td><td>Barrier: Bias and determining what data to use to train AI (Patient mentioned that each social “factor” has different weights of importance, which when applied to an algorithm will have different outcomes. This could cause a bias depending on how we weigh each factor).</td><td>Unbiased Training Data
Prevent Bias through Frequent Re-assessment</td><td rowspan="7">Bias and determining what data to use to train AI
- Discussed how each factor have differing weights of importance, also mentioned how some parameters could be biased toward (smoking) based on these weights, and providers were concerned about missing data and how that may influence the risk stratification results

Accuracy of AI tools
- Would risk predictions be accurate?

Safety Implications of AI tools
- If inaccurate predictions are made, would patients who need the most help actually be selected? What are the safety implications of this?

Provider burnout
- Specifically discussed the idea that clerical and admin staff are also burnt out, and we should not continue to load them with more tasks</td></tr>
<tr><td>Barrier: Provider Burnout (worried that providers do not have time to call “at-risk” patients)</td><td>Co-design/Co-collaborate
Value Proposition
Ease into the Technology</td></tr>
<tr><td>Barrier: Accuracy of AI tool (provider feels as though the social data collected would be insufficient to accurately deduce conclusions and predictions from)</td><td>Transparency</td></tr>
<tr><td>Barrier: Provider Burnout (feels like it would be nice to be able to be proactive and predict events, but also feel very limited for time already)</td><td>Co-design/Co-collaborate
Value Proposition
Ease into the Technology</td></tr>
<tr><td>Barrier: Bias and determining what data to use to train AI (believes if patients is a smoker, they will be automatically flagged over those who are not smokers)</td><td>Unbiased Training Data
Prevent Bias through Frequent Re-assessment</td></tr>
<tr><td>Barrier: Bias and determining what data to use to train AI, ALSO Barrier: Data structure (with incomplete and inaccurate data, we will likely bias toward the patients who have complete records and may miss those that don’t. garbage in = garbage out)</td><td>Unbiased Training Data
Prevent Bias through Frequent Re-assessment</td></tr>
<tr><td></td><td>Facilitator: Co-design/co-collaborate (ensure all stakeholders are at the table to design a tool that makes sense for THEM (ie. A nudge) instead of viewing the issue from a paternalistic/physician-centered-view. Also, patients can then be empowered to manage their own health)</td></tr>
</table>

	Barrier: Safety of AI tool (patient worried that AI tool may "miss" some folks who need care – especially if they are in the grey area/ borderline)	Reflect on Current State<br>Imperfections<br>Transparency<br>Value Proposition<br>Ease into the Technology	
	Barrier: Equity (those who are not as technologically-savvy may be "freaked out" by an AI nudge)	Deliberate Design<br>AI Literacy<br>Co-design/Co-collaborate<br>Ease into the Technology<br>Value Proposition	
	Barrier: Algorithm transparency (patients may be worried about where the data is coming from)	Transparency<br>National Rules & Regulations	
	Barrier: Informed consent (informed consent might help mitigate the above concerns regarding not knowing where the data is coming from, where it is stored etc.)	Facilitator: Co-design/co-collaborate: Ensure all stakeholders are at the table (Patients and providers should collaborate to design this initiative so that the nudges are well received)<br><br>Transparency<br>National Rules & Regulations	
	Barrier: AI Literacy (fearful of unknown big databases and AI, especially if it is telling them something negative about their health or risk of health)	AI Literacy	
**Use Case #5 (A Chronic Care Management Tool)** AI researchers at an American university developed an AI tool that uses deep learning to help primary care providers and patients manage chronic conditions. The tools analyzes data entered by patients in an app; it can also integrate with certain smart watch models. It sends alerts to providers when it detects something unusual. The researchers started a company to sell this tool and have approached several Canadian primary care clinics.	Barrier: Liability (participant mentions that patients should have autonomy to override tool alerts/ decide what is sent to care provider and what are the medical-legal liabilities related to this)	Transparency	- Patient anxiety from increased self-monitoring/too many alerts (explanation regarding the fact that sometimes medicine has guidelines, and patients may be permissibly above or below these guidelines. How would AI know to allow it? Can we calibrate these tools? Would we have to calibrate each and every tool for each and every patient?)- Meeting patient priorities when implementing (participant finds that insights and reports from self-tracking tools are not always meaningful. The tools need to be useful, meaningful and clinically relevant to the PATIENT).- Equity: some physicians worry that patients who are unable to use technology or self-monitoring would be at a disadvantage- Discussed interoperability and integration requirements with EMR system (physician concern)- Inappropriate use of patient data and security/ privacy risks - Concerned that interface must be patient friendly and not use jargon- Alert
	Barrier: Patient anxiety from increased self-monitoring/too many alerts (may be annoying if everytime your blood sugar was out of range, your doctor was informed and called)	Co-design/Co-collaborate<br>Deliberate Design	
	Barrier: Meeting patient priorities when implementing (participant finds that insights and reports from self-tracking tools are not always meaningful)	Co-design/Co-collaborate<br>Implement Iteratively	
	Barrier: Equity	National Rules & Regulations<br>Create Adoption Strategy<br>Co-design/Co-collaborate	

	Barrier: Adoption/Scalability/System Readiness<br><br>Barrier: Meeting patient priorities when implementing<br><br>Barrier: Interoperability<br><br>(would new tools seamlessly integrate with EMR? Would the insights produced be meaningful to the patient and/or physician or just a nuisance?)	Tech Infrastructure & Architecture<br>Interoperability<br>Leadership Commitment<br>Deliberate Design<br>Create Adoption Strategy	fatigue for the providers- Discussed what "valuable insights" would be important- Participant thought that these tools would not be useful in primary care, but would be better suited to acute care, palliative care, pain management, cancer)
	Barrier: Inappropriate use of patient data - Concern about monetization<br><br>Barrier: Security/ Privacy Risks<br><br>(concerned of privacy around data, and where it is allowed to be used or taken advantage of).	National Rules & Regulations<br>Transparency<br>Ethics Training	
	Barrier: Human-computer interface/usability(outputs should be broken down based on the audience they are designed for so that they can understand the importance and significance of the predictions/insights)	Co-design/Co-collaborateImplement Iteratively	
	Barrier: Alert fatigue (worry that tech may produce too many alerts causing important alerts to be missed)	Deliberate Design<br>Learn from Pilot Projects<br>Implement Iteratively	
	Barrier: Provider burnout (from all the data produced by wearable technology, causes provider burnout and "compassion fatigue")	Ease into the Technology<br>Co-design/Co-collaborate<br>Implement Iteratively<br>Deliberate Design<br>Learn from Pilot Projects<br>Create Adoption Strategy	
	Barrier: Patient anxiety from increased self-monitoring/too many alerts (may prevent chronic disease patients from being able to live in the present moment… this provider sees these tools as more effective in acute care, oncology, palliative care, and pain management.)	Deliberate Design<br>Learn from Pilot Projects<br>Implement Iteratively	

	Barrier: Impact to Healthcare Costs and Resources (some health resources may be wasted when acting on “false alarms” created by AI devices)	Leadership Commitment<br>National Rules & Regulations<br>Design Regulations based on Level of Risk<br>Reflect on Current State Imperfections	
**Use Case #1 (Risk Stratification Tool)** A large primary care organization that serves 36,000 people is using a new AI tool. It processes the usual health data found in EMRs and combines it with social data that the organization has collected on patients, like their housing, income and employment status, and their language preference. Clinic leadership has asked physicians to spend dedicated time each shift focusing on their top 10 patients who are at risk of getting sick.	Barrier: Impact to Healthcare Costs and ResourcesBarrier: Provider Burnout/ Workflow Interruptions(AI tool may add work for physicians where time is already tight. Must think about the impact to the user’s day otherwise it may cause inefficiencies/wasted effort and money in the system)	Co-design/Co-collaborateValue PropositionEase into the Technology	- Began discussion talking about how the AI tool may add work for physicians where time is already tight. Must think about the impact to the user’s day otherwise it may cause inefficiencies/wasted effort and money in the system- Concern for bias being introduced into the risk stratifying process (ie. based on language or who seeks care most often)- Equity (considerations around certain socio-economic parameters will increase one’s likelihood of being contacted, even if they are very healthy, participant believes these tools are more powerful on a population/epidemiological level and less on an individual level).
	Barrier: Bias (potential to have bias based on language or other social variables depending on who seeks care most often)	Prevent Bias through Frequent Re-assessment<br>Unbiased Training Data	
	Barrier: Algorithmic Transparency<br><br>Barrier: Safety of AI tool<br><br>(worried that without a clear criteria, patients who are complex may not get the help they need, not sure what variables/criteria determine if a person is high risk or not. Also, worried that if a patient is labelled as “complex” and physiscans don’t want to see them, the algorithm will learn not to prioritize them… worried about safety of the tool)	Transparency<br>Co-design/Co-collaborate<br>National Rules & Regulations<br>Implement Iteratively<br>Design Regulations based on Level of Risk	
	Barrier: Equity (considerations around certain socio-economic parameters will increase one’s likelihood of being contacted, even if they are very healthy, participant believes these tools are more powerful on a population/epidemiological level and less on an individual level).	Transparency<br>Prevent Bias through Frequent Re-assessment<br>Ethics Training	
**Use Case #4 (Triage)** An EMR vendor has developed a new AI-enabled tool. The vendor claims the tool can optimize patient appointment booking to help get those most in need seen first by their provider. The tool has a smartphone app that patients can use to book appointments and	Barrier: Equity (some patient populations may not want to interact with AI tools and may further deter them from the system as a whole)	Transparency<br>Prevent Bias through Frequent Re-assessment<br>Value Proposition	- Desire to have tool account for the whole person, not just their symptoms (holistic approach), and compare it to patient’s OWN baseline- Equity concerns arose from case; what if patient’s were not tech-savvy or did not own tech to complete the triage questions? Worried that this could further deter vulnerable populations from accessing the health system (PR-4404)- Gender bias addressed through example with Babylon App predicting different outcomes in male and female heart attacks
	Barrier: Bias (example of how a previous tool, Babylon App, recommended different diagnoses to men and women who were presenting with similar symptoms)	Prevent Bias through Frequent Re-assessment<br>Unbiased Training Data	

describe the reasons for the visit. The vendor offers clinics that use their EMR system free use of the tool for one year.	Barrier: Trust in the AI tool (Providers worried that those who actually need to be seen may be triaged lower based on their attitudes and language compared to hypochondriacs that know how to speak the right language to get in right away. AI may not be able to decipher the difference).	Transparency<br>Co-design/Co-collaborate<br>National Rules & Regulations<br>Implement Iteratively<br>Design Regulations based on Level of Risk<br>Reflect on Current State Imperfections	based on same symptoms and different genders- How will an AI tool know how important a given concern is to a patient? Some people are very bothered by a non-concerning symptom, while others are not bothered by concerning symptoms- Additionally, discussed the idea that many patients know the critical words to use to be seen quickly, and may know how to outsmart an AI triage tool to be ranked higher on the list (hypochondriac vs. anti-hypochondriac discussion)- Emphasis on including clerical folks at the table during co-design process as they have unique information about patient populations and the questions that should be asked (i.e. a farmer making an appointment during harvest)- Concerned about the criteria being used to arrive at the triage answer (algorithm transparency)
		Facilitator: Co-design (include clerical/admin staff in the design process so that they can provide their insights into the variables that may help guide a tool like this triage use-case. They may have specific thought patterns based on the patient population they work with.)	
	Barrier: Safety of AI tool (what if there is an emergency that calls in and the AI tool does not recognize this?)<br><br>Maybe also:<br>~~Barrier: Concern about AI taking over jobs (replacing a knowledgeable person that can ask more questions with an AI tool)~~	Transparency<br>Co-design/Co-collaborate<br>National Rules & Regulations<br>Implement Iteratively<br>Design Regulations based on Level of Risk<br>Reflect on Current State Imperfections	
	Barrier: Algorithm transparency (concerned about what criteria the tool uses to determine triage list)	Transparency	

2. Final List of Codes (agreed upon by KD, TLU, AP)

Barriers/Challenges
Security/ Privacy Risks
Informed Consent
Patient-Provider Relationship
Trust in Provider
Provider/End-user Training
Provider Burnout/ Workflow Interruptions
Liability
Algorithmic Transparency (what parameters are being used to produce a given diagnosis?)
Bias and determining what data to use to train AI
Adoption/Scalability/System Readiness
Lack of Regulation, Governance, & Oversight
Inappropriate use of patient data (Concern about monetization (insurance etc.))
Data storage and ownership
Impact to Healthcare Costs and Resources
Equity
Accuracy of AI tool
Safety of AI tool
Trust in AI tool / technology
Concern of de-skilling/dependency on tool
AI Literacy
Lack of interoperability between different applications
Human-computer interface/usability
Patient anxiety from increased self-monitoring/too many alerts/patient burnout
Meeting patient priorities when implementing
Ownership & Control of AI tool- Concern over private companies providing and controlling the technology
Data structure (how will we address the fact that providers enter information into their systems differently?)
Inter-Jurisdictional Barriers (Hard to work nationally, when healthcare is regulated provincially)

Facilitators/Opportunities/Enablers to Overcome Barriers	
**Code**	**Definition**
Value Proposition	Create a clear value proposition for each user/person affected by technology (what's in it for them? What benefits will they see from this technology?)
Ease into the Technology	Start with AI technology that can complete basic operational tasks before transitioning into the implementation of complex AI systems
Transparency	Outline all of the benefits and limitations of the technology to all affected and communicate these to the users to manage expectations
Co-design/Co-collaborate	Ensure all stakeholders are at the table during both the design and implementation of these tools. Include representative stakeholders from different communities and different types of users (administrative clerks, providers, patients etc.)
Design and implement iteratively	Use iterative design and implementation strategies to allow for stakeholder feedback throughout. This will ensure the tool is designed for those that it will affect (and not the agenda of a private business)
Interoperability	Invest in interoperability to enable different technological systems to "talk to one another" thereby enabling integration between clinics and hospitals
Unbiased Training Data	Use unbiased, representative, good quality data to feed algorithms
Prevent Bias through Frequent Re-assessment	Ensure AI tool remains free of bias overtime by conducting routine re-assessments and analyses of the tools
Tech Infrastructure and Architecture	Organize tech infrastructure and architecture to support AI tools
Leadership Commitment	Encourage health leaders and government players to demonstrate commitment to AI (lead by example)
National Rules & Regulations	Create and implement national AI rules and regulations for healthcare (there is a need for content standards and protocols to further enable interoperability, and a need for regulatory standards to ensure representation and bias mitigation within the algorithms and the training data sets)
Design Regulations based on Level of Risk	Create regulations based on level of risk that the tool may pose to its users. For example, there may be different regulations for tools that conduct administrative-related functions than those that are designed for clinical decision making and diagnostic functions
AI Literacy	Provide education and transparency into what AI tools are and how they are used in order to reduce concern of the unknown and enable all stakeholders to participate in co-development conversations
Deliberate Design	Deliberately select AI technologies to align with what we want humans to do versus what we want technology to do. There is a need to be deliberate and weigh the benefits and risks before implementing AI technologies
Learn from Pilot Projects	Use pilot projects as examples to learn from
Reflect on Current State Imperfections	Recognize the fact that medicine is not perfect today. Humankind makes mistakes, adverse events occur, and diagnostic errors transpire. Consider comparing the new rates of errors/adverse events with the AI to the current rate.
Ethics Training	Consider designing ethics training courses for all of the stakeholders who will be involved in the design, testing, implementation, and use of AI tools.
Create a Robust Adoption Strategy	Arrange for adoption strategy where providers can slowly ease into using the technology. It may begin with tighter constraints at the start, and overtime as trust is built, it can be given more autonomy. (staged approach). This strategy should also be versatile enough to be applicable to many different areas of primary care clinics.

3. Code Frequency (Upon Re-analysis)

	R1	R2	R3	R4	R5	R6	SUM
Security/ Privacy Risks	1	2	0	0	1	0	4
Informed Consent	2	0	0	2	0	0	4
Patient-Provider Relationship	0	1	2	2	0	3	8
Trust in Provider	0	1	2	0	0	0	3
Provider/End-user Training	0	0	1	0	0	0	1
Provider Burnout/ Workflow Interruptions	6	3	0	0	0	1	10
Liability	0	1	0	0	0	0	1
Algorithmic Transparency (what parameters are being used to produce a given diagnosis?)	3	1	1	0	1	1	7
Bias and determining what data to use to train AI	3	3	1	1	3	1	12
Adoption/Scalability/System Readiness	0	5	0	1	2	1	9
Lack of Regulation, Governance, & Oversight	0	0	0	0	0	0	0
Inappropriate use of patient data (Concern about monetization (insurance etc.))	0	3	0	0	1	0	4
Data storage and ownership	1	0	0	0	1	1	3
Impact to Healthcare Costs and Resources	1	4	0	0	0	0	5
Equity	1	3	1	2	3	0	10
Accuracy of AI tool	2	0	0	1	2	0	5
Safety of AI tool	2	2	1	0	0	0	5
Trust in AI tool / technology	0	1	3	0	1	0	5
Concern of de-skilling/dependency on tool	0	1	0	0	0	0	1
AI Literacy	3	0	0	0	0	0	3
Lack of interoperability between different applications	1	1	0	1	0	1	4
Human-computer interface/usability	0	3	0	0	1	0	4
Patient anxiety from increased self-monitoring/too many alerts/patient burnout	0	5	0	1	0	2	8
Meeting patient priorities when implementing	0	5	0	0	0	0	5
Ownership & Control of AI tool- Concern over private companies providing and controlling the technology	0	0	0	2	1	1	4
Data structure (how will we address the fact that providers enter information into their systems differently?)	0	0	0	1	2	0	3
Inter-Jursidictional Barriers (Hard to work nationally, when healthcare is regulated provincially)	0	0	0	0	0	1	1
