# Supplementary material for: Implementing artificial intelligence in Canadian primary care: Barriers and strategies identified through a national deliberative dialogue
Source: PLoS One. 2023 Feb 27;18(2):e0281733. doi: 10.1371/journal.pone.0281733 (PMC9970060; doi:10.1371/journal.pone.0281733)
Supplement: S1 Appendix — (DOCX) [file pone.0281733.s004.docx]

***Future Perspectives on AI in Canadian Primary Care:***

**A National Deliberative Dialogue Series**

**September 9^th^ – October 15^th^, 2020**

**PARTICIPANT INFORMATIONAL MODULE**

**Prepared by Tara Upshaw, MHSc student**

Before distribution, this module was reviewed by four content experts (CSG, TCYC, JG, ADP), three members of the public and a patient partner.

**Table of Contents**

| **1 \|** | Introduction……………………………………………………….... | 27 |
| --- | --- | --- |
| **2 \|** | Deliberative dialogues: what to expect…………………………. | 28 |
| **3 \|** | Artificial intelligence: an overview……………………………….. | 30 |
|  | Machine learning…………………………………………… | 32 |
|  | Deep learning………………………………………………. | 33 |
|  | Other AI methods…………………………………………. | 35 |
| **4 \|** | AI in medicine and healthcare…………………………………… | 37 |
| **5 \|** | Applying AI in primary care………………………………………. | 39 |
| **6 \|** | Ethical considerations when applying AI to health data………. | 43 |
| **7 \|** | Summary…………………………………………………………… | 45 |
| **8 \|** | Pre-session questionnaire……………………………………...... | 46 |
| References……………………………………………………………… | | 47 |
| Glossary……………………………………………………………….... | | 49 |

1. **Introduction**

***What is the purpose of this module***?  You have received this module because you are participating in the research study: **Patient, provider, and health system leader perspectives on artificial intelligence technology in primary care.** You do not need to know anything about artificial intelligence (AI) to participate in this study. This module is meant to teach you about AI and its possible uses in primary care so that you can feel comfortable talking with other participants.

You are not expected to be an expert on AI, and you will likely have further questions after reading this module. We encourage you to bring these questions to your sessions, along with your views and ideas.

This module will:

- Describe the design of the study and what to expect during your sessions
- Define AI and describe computer programming methods that underly AI technologies
- Explore applications of AI in medicine broadly
- Describe possible applications of AI in primary healthcare
- Describe ethical considerations when applying AI in the context of healthcare

***Where does this information come from?*** A variety of sources, including academic literature and consultations with experts in health information technology and AI. A reference list and a glossary of **bolded blue** terms is available at the end of this document.

***How long will this module take to complete?*** This module will take most people about 30 minutes to read. There is a short questionnaire at the end that will take up to 5 minutes to fill out. This questionnaire must be completed online.

***How does the module work?*** The online module is delivered using a secure survey platform. You received a custom link that saves your progress and tracks your questionnaire responses. Since you have chosen to review the printable PDF version, please remember to return to this link to complete the questionnaire when you’re finished.

1. **Deliberative dialogues: What to expect**

***What is a deliberative dialogue?*** A **deliberative dialogue** is a discussion among people involved in or affected by future decisions about a high-priority issue. A dialogue aims to bring stakeholders together to understand a topic in greater depth and share different views.^53^ Each dialogue will involve up to 10 participants. Dialogue participants have an opportunity to identify what is important to them about an issue and work together with other participants to provide advice to researchers and policymakers. Results may include consensus recommendations and emphasis on areas of disagreement among participants.

***What is primary care?***  **Primary care** refers to the services involved in health promotion, illness and injury prevention, and the diagnosis and treatment of illness and injury.^54^ Primary *healthcare* is usually the point of first contact with a healthcare provider when you are sick or injured. Primary care providers include family physicians, nurse practitioners, pharmacists, and others that contribute to your holistic well-being, including social workers, dieticians, and traditional healers. Primary care providers help you move through the health system if you need more specialized care or enrol in social programs that can support your health.

***Why is a deliberative dialogue about the future of AI in primary care important?*** Advances in AI are driving innovation in most industries, including healthcare. It is expected that AI *will* change primary care and other areas of healthcare soon.^55^ Exactly how AI will change primary care is unclear because it is not widely used in most healthcare settings. Many factors will interact in complex ways, including primary care provider roles, patient preferences, policies, political considerations, and commercial interests. What’s most important for the success of any AI-based technology is that its *function* matches the *needs* of patients, providers, and health administrators.

This deliberative dialogue series will help us understand how AI can be useful for people who use and work in primary care. Dialogues will allow us to anticipate how certain uses of AI will impact patient experience and clinic workflows. As well, the dialogues will help us form recommendations for policymakers and identify critical unanswered questions to address through future research.

***Who is involved in this dialogue?***  Patients and providers from across Canada were invited to reflect diverse life and work experiences. Policymakers in all provinces and territories involved in shaping policies that affect how AI is used in health settings were invited to participate. Most participants will take part in more than one round of the study.

***What will happen during the dialogues?*** Deliberative dialogues will occur over four rounds (Figure 1). Each round, a trained facilitator will work with participants to focus on a different aspect of AI in primary healthcare. Participants will receive short summaries of the previous dialogue rounds before their session. These summaries may include new information not included in this module that becomes relevant as the dialogues progress.

***
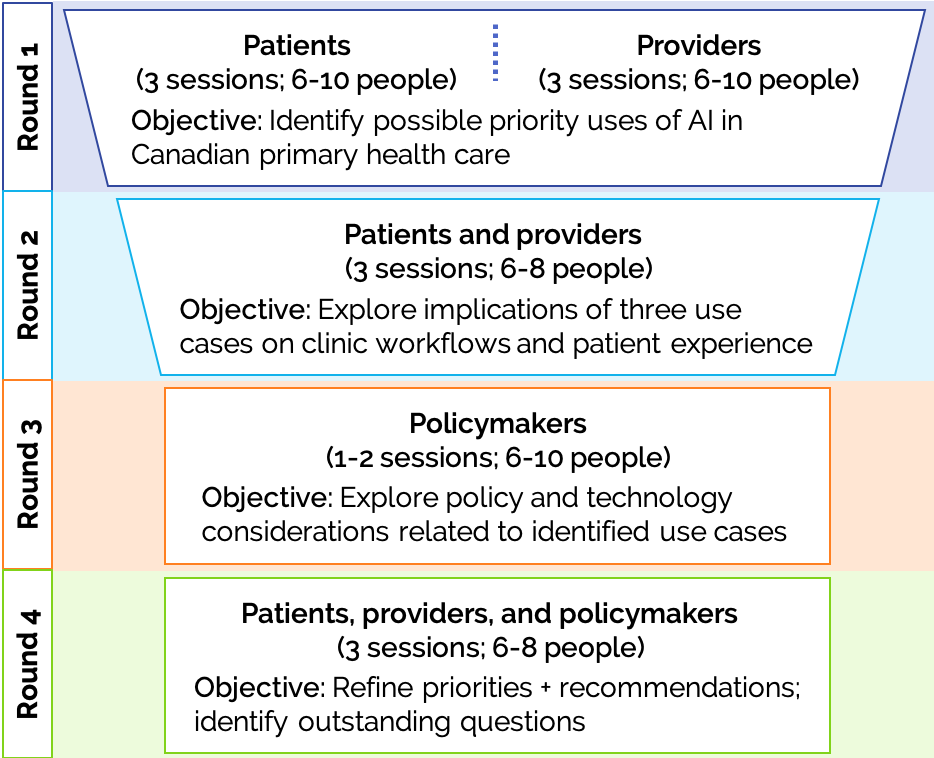
***

Figure 1. Session topics for Future Perspectives on AI in Canadian Primary Care dialogue series

In *Round 1*, primary care patients and providers will meet in separate groups to discuss the application of AI in primary care and identify possible priority uses.

In *Round 2*, patients and providers will meet *together* to explore three use cases “prioritized” in the previous round. Discussions will focus on the impact that an application may have on patient experience and clinical workflows. With help from the facilitator, participants may form preliminary recommendations for policymakers and researchers.

In *Round 3*, policymakers from across Canada will meet to discuss key findings from Rounds 1 and 2 and provide insight into relevant policy contexts.

In *Round 4*, representatives from all three participant groups will meet together to integrate Round 3 input from policymakers. Participants may adjust priority uses, revise or finalize recommendations, or highlight important areas of disagreement.

***What is my role as a participant?***As a participant, your role is to bring your unique perspective to the discussion and learn from others' perspectives. The facilitator may ask participants to develop the reasoning behind their views and ideas. The information you read and hear may inform your opinions, and your opinion might (or might not) change over time. A dialogue is about embracing diverse views among us and finding ways we can work together to guide future directions.

***What is the role of others?***

**Facilitator** The facilitator will work with participants to:

- - 1. Discuss questions prepared by the research team and questions that emerge during the discussion
    2. Help participants form recommendations.
    3. Respectfully explore areas of disagreement.

**Observer** A research staff member will attend all sessions to take notes on the discussion and support session logistics.

***Are my contributions to dialogues confidential?***  Participants are free to use the *information* gained during dialogues. We ask that all participants not disclose the identity or the affiliation of a speaker. In other words, it’s ok to talk about the dialogues with people who didn’t take part, but we can't credit anything we hear to a specific person or the place they work.

All participants will only be asked to identify themselves by their first name and their participant type (*i.e.* patient, provider, or policymaker) during the session. We will provide instructions for setting your username on Zoom.

Sessions will be audio-video recorded. Recordings will be password-protected and stored on secure servers at St. Michael’s Hospital. Only members of the study team will have access to recordings. Any identifying information will be removed during analysis.

1. **Artificial intelligence: an overview**

In this section, we will define artificial intelligence (AI) and review common computer programming methods used to create most everyday AI-based applications. By the end of this section, you should understand how deep learning methods fit within AI, and how AI relates to Big Data (Figure 2). Don’t worry about memorizing details. Focus on the big picture and use the glossary if you need to jog your memory on specific terms.


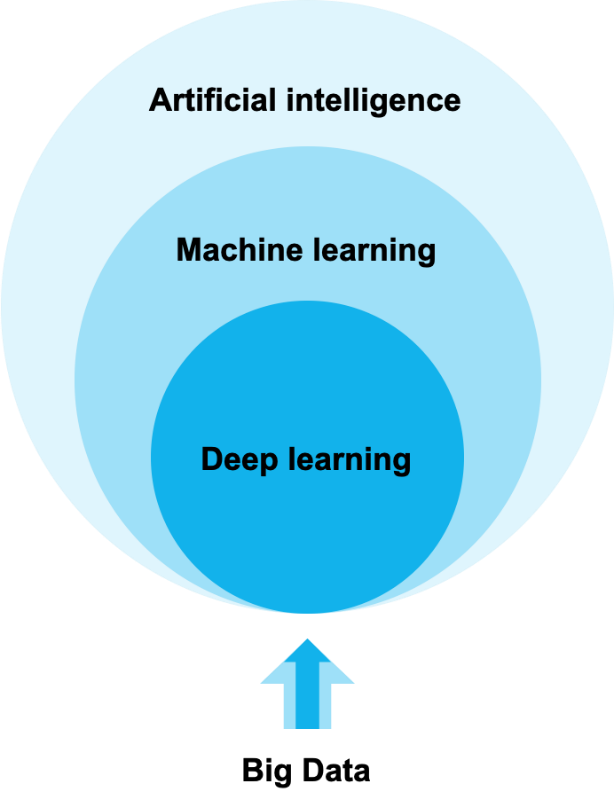


Figure 2. Artificial Intelligence, Machine Learning, and Big Data (adapted from Jillian Macklin)

We will use the terms ‘algorithm’, ‘system’, ‘program’ and ‘method’ interchangeably when talking about AI. AI methods are computer algorithms. An **algorithm** is a set of step-by-step instructions for solving a problem. A computer algorithm can be as simple (e.g. If it’s Saturday at 9 AM, send a reminder) or complex (e.g. Identify pedestrians).^56^ We will learn more below about how AI algorithms can be combined to produce applications with complex “intelligent” functions.

***What is artificial intelligence?*Artificial intelligence** is a broad field of science concerned with getting computers to do tasks that would normally require human intelligence. In this definition, intelligence refers to processing information, reasoning and learning, planning actions, and communicating in natural language. Another way to think about AI is as a *general-purpose prediction technology* that estimates missing information from available information.^57^

Alan Turing established the concept of AI in 1954.^58^ Since then, computer scientists have developed a variety of methods that allow computers to mimic human intelligence. These methods can be grouped into several types; each type is suited to particular intelligent tasks. A combination of methods is usually used when developing AI technologies.

Today’s AI applications are "narrow." **Narrow AI** programs can only do what they were designed to do.^59^ For example, an AI program that can beat a human in a chess match can't solve a complicated math problem. "Narrow" AI applications are often better than humans at the tasks they were designed for but cannot develop additional skills without being programmed by humans.

In contrast, **general AI** refers to a single system that can learn in different situations and then apply broad knowledge to solve any kind of problem – like the human mind!^59^ This is what many people think of when they hear "AI." There are currently no actual examples of general AI. This is not surprising when we consider the complexity of human learning and problem-solving.

***What is Big Data? How is it related to AI?*** “Big Data” is a term used to describe data produced by a variety of sources in large volumes at a fast pace. Big Data has grown across industries in the last three decades because of advances in computer processing power and storage, widespread adoption of mobile devices, and increased internet availability.^60^ AI methods allow us to condense and make sense of these datasets. Advances in machine and deep learning in the last 10 years have depended on the availability of large, labelled “Big” datasets.

A **dataset** is a collection of data gathered using the same criteria for a specific purpose. Datasets from different sources can be shared and combined to create **linked datasets.** Linked datasets contain a broad range of information that can be analyzed to shed light on complex issues. When linked data are used for research purposes, they are **de-identified** to reduce the possibility that they could be traced back to any individual.

For example, the Insurance Corporation of British Columbia (ICBC) collects data from individual drivers in BC each time a person renews their car insurance. The Medical Service Plan collects data on visits to physicians in British Columbia. These two datasets could be shared and combined to reveal relationships between health care use and driving practices.^61^ As information technology continues to develop, new sources of data will appear, allowing new linked datasets to be created. New linked datasets offer new opportunities for research in many areas, including AI.^61^ We might explore data linkage in more detail during Round 2 of the study.

The following sections describe some common AI methods that form the basis of most modern AI applications. All of these methods work best when applied to Big Data.

**3.1  Machine learning**

***What is machine learning?*Machine learning** refers to AI methods where a computer program learns from experience over time.^60^

For example, algorithms that detect spam emails are “trained” through exposure to many examples of emails that have been manually *labelled* as spam or not spam. The spam detection algorithm will learn particular words or combinations of words that increase the chance that an email is spam. A feedback loop can be used to help the program improve after making a mistake.

The spam detection album is an example of **supervised learning**, in which labelled examples (in this case, emails labelled as spam or not spam) are included in a *training* dataset. In **unsupervised learning,** there are no labelled examples and an algorithm instead groups data by similarities. Unsupervised machine learning is increasingly used to drive discovery in basic science and medical research by revealing unexpected relationships among data. Both types of learning will test the performance of an algorithm on a *test* dataset that the algorithm has never analyzed before.^56^

As narrow AI, machine learning algorithms are usually more accurate than humans at the prediction tasks they are trained for. A machine learning AI system is not truly intelligent because it does not understand what it was trained to do. A spam detection algorithm can be great at filtering spam, but it will never understand *what* spam is and why it’s bad in the way humans do. If a new type of spam emerges, it will probably have to be retrained by a (human) computer programmer to recognize it.

Machine learning forms the basis of most AI systems.

**3.2 Deep learning**

***What is deep learning?* Deep learning** refers to a subtype of machine learning methods that identify hidden and complex patterns in a dataset. These patterns can be used to classify new data into a defined category (*i.e.* using supervised learning methods) or group new data by similarity (*i.e.* using unsupervised learning methods) with very high accuracy.^4^

Deep learning is a more recent type of AI that has improved existing technologies and helped create others that were not possible before, like self-driving cars. The distinguishing characteristic of a “deep” learning algorithm is the use of many layers. Each layer identifies different features of the data set; combining the layers allows for detection of complex patterns that can be used to make very accurate predictions. With “shallow” AI methods like classic machine learning, scientists must spend time:

1. identifying data features important for making accurate predictions, and
2. manually transforming these features into math a computer understands.

Deep machine learning is powerful because it eliminates the need for manual feature selection and representation. This allows for much more complex prediction tasks.

Consider the example of distinguishing photos of cats from dogs – a popular project for AI developers-in-training. This initially seems like a simple task because we have been doing it all our lives – but think about what makes a dog *visually* different from a cat. Both have four legs, two eyes, and two ears - all roughly in the same position. Both have a tail and come in different colours. Dogs and cats can be similar in size.

So, *how do we know they are not the same?* Looking closely at the photos above, you will find no hard-and-fast rules for deciding. Yet, we can still tell the difference, but it's hard to pinpoint how we do it. Most of us will say that it has something to do with a subtle pattern of features that exist uniquely together. We have learned these patterns over time and by exposure to examples, and probably by making a few mistakes. As you can imagine, these visual relationships are extremely difficult to represent in computer language.

Deep learning methods solve this problem by automatically detecting features of a dataset and defining relationships between them in mathematical terms a computer understands. This mimics the nuanced and highly accurate process of human pattern recognition. Humans can learn to categorize or group objects pretty well from a small number of examples. Deep learning algorithms learn from thousands or even millions of examples, making them extremely accurate at the prediction task they are designed for.

It is important to note that the “features” that a deep learning algorithm extracts from a dataset are abstract mathematical *representations* of data qualities. They are not always concrete qualities that we can easily name. This can make it difficult to precisely understand how a deep learning AI system arrives at a prediction, even when it is correct. This is called **non-explainable AI**. For this reason, deep learning algorithms are often compared to a “black box.”^4^

Deep learning is well-suited to prediction problems in medicine because health and disease often involve complex interactions. As you can imagine, it is sometimes necessary for clinicians to understand contributing factors to an illness or injury to inform effective prevention and treatment. Efforts to develop “explainable” deep learning systems are ongoing.

**3.3 Other AI methods**

**Natural language processing** (NLP) refers to AI methods used to interpret human communication and reproduce it in various forms.^56^ In combination with deep learning, natural language processing is the basis of automated translation services like Google Translate, chatbots, and virtual personal assistants such as Apple’s *Siri* and Amazon’s *Alexa.* Most advanced NLP systems also use deep learning.

**Computer vision** refers to AI algorithms that interpret digital images or videos.^4^ Computer vision underlies facial recognition software and autonomous vehicles. Modern computer vision systems almost always use deep learning to perform their function.

For example, social media platforms like Instagram, Snapchat, Facebook, and TikTok use computer vision for their video filter technology. Filters use an AI algorithm to detect the features of a digital image that represent a human face. An animated filter is then applied to the face and can follow that face's movement in the frame.

Cognitive analytics and robotics are other types of AI we will not discuss in depth for this study.

**If you would like to take a break and return to this booklet later, this is a good place to pause.**

1. **AI in medicine and healthcare**

As we have learned, AI is not so much one thing as a set of computer programming methods for analyzing large volumes of data from various sources to identify patterns. Patterns within data can be used to make highly accurate predictions. Predictions can add value to a wide range of tasks or problems in most industries, including healthcare.

Several factors are driving the adoption of AI in healthcare today.^3^ First, labelled digital health datasets have grown exponentially in the last 30 years. This is the combined result of:

- Development of comprehensive **administrative datasets** that track a patient’s journey through a healthcare system
- Widespread adoption of **electronic medical records** (*i.e.* the computer software your doctor enters notes into during a visit)
- Advances in medical imaging technology
- Decreased costs of genomics technology (*i.e.* gene sequencing and mapping) resulting in more **genomic data**
- Broad uptake of “wearable” devices that track **physical data** like your heart rate, body mass index or your blood glucose levels

These datasets can potentially be linked to provide a detailed data profile of an individual or a population. Second, faster hardware emerged in the mid-2000s that allowed for the computational power necessary to analyze such large and high-variety datasets using deep learning.

Clinical application of AI (especially deep learning) has been most rapid in medical disciplines that rely heavily on medical imaging. In many cases, deep learning AI systems outperform individual clinicians when interpreting medical images. For example, a group of AI scientists at Stanford University in California developed an AI algorithm to diagnose skin cancer from pictures of abnormal skin marks using deep learning and computer vision methods.^62^ The algorithm was trained on 129,450 images of skin conditions labelled as cancer or not cancer by dermatologists. Algorithm performance correctly classifying skin cancer was compared to the performance of 21 dermatologists. Algorithm accuracy was equal to or better than that of most dermatologists involved in the study. To our knowledge, this tool has not yet been tested in clinical settings.

Increasingly, healthcare organizations are interested in applying deep learning AI to develop **risk stratification** tools that can organize patients according to their risk level for a health event.^63^ These tools can be used to inform preventive care and support hospital operations (*e.g.* staffing changes when care needs will likely be high). When implemented in clinical practice, a provider may increase support for individuals at increased risk for a particular event, shifting healthcare resources to those predicted by an algorithm to have the most need.

For example, a group of AI researchers at the University of California applied deep learning AI to raw data from patient electronic medical records to predict hospital readmission and death.^64^ The algorithm was tested on two hospital electronic medical records datasets and predicted events more accurately than existing non-AI predictive models. Other researchers have developed tools that can predict blood infection risk or delirium during a hospital stay, among many other examples.

It’s important to note that few algorithms developed by AI researchers have been widely implemented in clinical practice. This limits our understanding of how well prototype tools integrate within existing healthcare delivery systems or improve patient health.

1. **Applying AI in primary healthcare**

***What kind of data is created through primary care?* Electronic medical records** **(EMRs)** are the richest source of data in primary care. In Canada, more than 70% of primary care settings in most provinces collect and store patient data in electronic health records.^65^ EMRs contain **clinical data** – data collected by a hospital or provider in order to provide appropriate healthcare services. Clinical data include

- sociodemographic information, such as your age, race or ethnicity, gender, or income level
- laboratory and medical imagining test results, and
- unstructured notes your provider makes during your visit. These notes may include descriptions of your symptoms, existing health conditions, or even important life events, such as the loss of a loved one.

EMRs also contain your provincial health insurance number. EMRs can be linked with administrative data maintained by your provincial health authority that records your health system encounters. It is now possible to sync vital sign or activity data from wearable devices (*e.g.* Apple Watch of Fitbit) with many EMR systems.

This wealth of data is difficult to condense and analyze for use during a clinic visit. The AI methods we have discussed can be applied to create value for primary care patients and providers.

***Is primary care ready for AI?***  As with other medical disciplines, there are currently few examples of *widely* used AI tools in primary care. Searching online, you will find many tools developed by private companies that could be used in primary care. Some of these are likely already in use in some primary care settings. For most of these tools, there is little academic research on their effectiveness in improving health.

Additionally, a 2020 review found that research specific to AI in primary care is in the early stages of maturity.^66^ Of 405 studies identified in the review, most were focused on developing or improving AI methods to achieve good algorithm performance, and not on how those tools function in a clinical setting. Only 14.1% of study teams included primary care providers. The authors of this review emphasized the need for research that:

1. Involves interdisciplinary research teams that include people with direct clinical experience
2. Engages end-users (*e.g.* patients, providers, or administrative staff) throughout the development of AI applications
3. Evaluates the effectiveness of AI-based tools for improving health when used in primary care settings

***What can AI do for primary care?*** It is not guaranteed that AI will transform primary care or any other areas of medicine. Sometimes new technology has unexpected consequences.

For example, EMRs were expected to massively increase efficiency for physicians. EMRs made it easier for providers to bill provincial health insurance plans for their services, but many providers find they have to spend more time typing to maintain the patient chart than interacting with their patients. This ‘4000-clicks-a-day’ problem has been linked to physician burnout.^67,68^ This problem exists because EMRs were designed to improve billing efficiency and not patient-centred care. What’s most important to any new technology's success is that its *function* matches the end-user’s needs.

Below are examples of ways AI may be applied to primary care data. Some of these examples are based on commercially available applications or prototype algorithms described in published research. Other examples are possible with today’s AI methods and existing primary care data sources but haven’t been created yet.

As you read, think about what use cases stand out to you. Which examples would most improve your experience as a patient or your job as a provider? Which ones don’t seem as useful? Can you think of other problems in primary care that could be addressed by AI? Can you anticipate issues that might arise if you had to use a particular application?

These are some of the questions that we will explore more in-depth throughout the deliberative dialogues. On the last page of this module, you will be asked to list some of the use cases that you think are important or interesting or other ideas you have about where AI could be applied in primary care.

| **Table 1. Potential uses of AI in primary care** | | |
| --- | --- | --- |
| **Category** | **Definition** | **Example** |
| Self-care, illness prevention and wellness | Tools that support people in living healthier lives | A machine learning algorithm analyzes vital sign data from a patient's smartwatch in real-time, documenting trends in their primary care EMR. They receive personalized reminders to exercise, eat well, and get enough sleep. Their physician is alerted when trends show a decline in heart health. |
| Triage and early diagnosis | Tools that help triage patients and identify the need for additional health resources | A machine learning-based symptom checker informs a patient with a gradual development of severe foot pain to book an appointment with their primary care provider as early as possible. |
| Diagnostics | Tools that assist providers with point-of-care diagnosis | A primary care provider uploads a cell phone photo taken of a patient’s retina to an app that uses deep learning to predict the risk of complications from diabetes. He refers the patient to an ophthalmologist.^69^ |
| Clinical decision support | Tools that structure relevant information to help physicians determine treatment course or need for referral to specialist or acute care services | An EMR-integrated machine learning algorithm predicts which patients are at high risk for becoming infected with HIV within a three-year timeframe. Risk profiles can help primary care providers who would most benefit from pre-exposure prophylaxis medications.^70^ |
| Care delivery | Tools that support direct interactions between patients and providers | A natural language processing tool automatically converts the conversation between a patient and provides into chart notes, orders laboratory tests, and writes referrals to specialists during a clinic visit. This tool can also reach out to patients in advance of the appointment to gather necessary information.^71^ |
| Chronic care management | Tools that help patients and providers manage chronic diseases like diabetes or heart disease | A patient with diabetes has a blood glucose monitor that syncs with an AI-based app on their phone. The algorithm learns the patient’s dietary and insulin delivery schedule over time. It begins to send helpful reminders to eat, check blood glucose, and inject insulin. The app is integrated with the patient’s primary care EMR. It notifies the provider when the patient’s insulin needs appear to change significantly. |
| Population health management | Tools that analyze large data sets to identify trends in population health to inform shifts in clinical programs and intervention targeting | A deep learning algorithm analyzes a clinic’s raw EMR data. It identifies the patients at the highest risk for hospital admission within the next 30 days. Providers in the clinic schedule appointments with these patients to discuss their health and preventive interventions.^64^ |
| Healthcare operations | Tools that decrease time spent on routine administrative tasks that occur in the background of patient care | A classical machine learning algorithm learns that times and days of the week where appointments are in highest demand, and helps clinic clerical staff optimize the staffing schedule |

1. **Ethical considerations when applying AI to health data**

AI has great potential to improve health and healthcare, but as with all innovations, there are limitations. These limitations raise some important ethical questions.

***What is bias? Why does it matter?***Many researchers have shown that non-health-related AI can worsen existing social inequalities by duplicating or worsening race, gender and other biases.^72^ **Bias** describes a preference for one thing, idea, person or group compared to another, usually in a way that is considered unfair.

It is important to realize that *AI algorithms are only as good as the data they are trained on*. Some groups of people with better access to healthcare may contribute to health datasets more than other groups. AI algorithms trained on datasets that poorly represent certain groups may make less accurate predictions for members of those groups.

When used in healthcare decision making, biased predictions can worsen health inequality between groups.

Consider the following hypothetical example:

| *A provincial health services agency partners with a private company to develop an AI algorithm that identifies patients at high risk of serious complications from their chronic diseases. The goal is to use this algorithm to target more supportive primary care resources to these patients to reduce their risk and improve their overall health.*  *The company links the primary care EMR data for all provincial residents with system administrative records, and applies explainable, supervised machine learning methods. The primary variable used for prediction is the historical costs of care. Patients who have previously received the most healthcare are the first to be targeted with new comprehensive care management programs. The company files a patent on the algorithm.*  *A few providers notice that most of the patients flagged as high-risk are White. They voice their concern to provincial health authority, which then hires an external group of scientists to evaluate the algorithm under a strict non-disclosure agreement. The scientists learn that most of the patients flagged by the algorithm are not the sickest patients in the province. Black and non-status Indigenous patients are sicker on-average than other racial groups, but data on sickness wasn’t included when training the model.*  *Adjusting for sickness, the researchers find that Black patients should be receiving 46.5% of comprehensive care program resources instead of the current 17.7%. Non-status Indigenous patients should receive 23.4%, instead of 4%. It is well-known that non-White people in Canada and the United States experience more barriers to accessing healthcare. This means that their overall healthcare costs are decreased compared to White people, even though their health needs are greater, on average.* |
| --- |

This example is based on a published scientific analysis of a real AI algorithm applied to 200 million people each year in the United States.^73^ This algorithm is not a unique case but represents a general approach to risk prediction in the health sector. If health systems use biased algorithms when distributing health resources, the health of certain groups will improve while the health of others will get worse. To combat bias, many researchers have emphasized the importance of including patients, clinicians, and ethicists from the beginning when developing AI applications for use in patient care.

***What other ethical considerations are there?***There are other ethical questions relevant to the above example. What about the patients whose data was used to train the model? Did they agree to have their data used for the development of the tool? Do they *have* to consent? How do you feel about your health data being used to train an AI algorithm used in other people's care?

Many private companies do not want to disclose their methods for developing a novel AI tool and file patents to protect this information. How can we trust that a tool marketed by a private company is unbiased and safe to use for *all* patients? Should developers and healthcare providers have to tell the public if they learn that an algorithm they are using is biased? What if a tool was instead developed and sold by researchers who published their methods in academic journals reviewed by other researchers?

And what about the **“black box”** quality of some deep learning AI systems? When is it important for the patient and provider to know what features of a dataset contribute to a prediction?

As you can see, many important ethical questions arise when we think about the use of AI tools in healthcare settings. There are currently no clear answers to many of these questions.

1. **Summary**

Artificial intelligence is a broad field of science concerned with getting computers to do tasks that would normally require human intelligence. AI methods can be applied to large datasets to identify relationships and extract meaning. These relationships can be used to sort new data into predefined categories or group data by similarity. Machine learning, deep learning, computer vision, and natural language processing are all types of AI that can be used alone or together to get a computer to perform a narrowly defined task. All of these methods work best when applied to large datasets.

Both growth in labelled digital health datasets and increases in computer processing power are driving the adoption of AI in healthcare today. Clinical application of AI has been most rapid in medical disciplines that rely most on medical imaging. There is growing interest in applying deep learning AI to develop risk stratification tools that can organize patients according to their risk level for a health event. To date, few algorithms developed by AI researchers or companies have been widely implemented in clinical practice. This limits our understanding of how well prototype tools integrate within existing healthcare delivery systems or improve health.

Primary care electronic medical records are a rich source of data that can be linked to other datasets, including data about health system encounters and data from wearable devices. The AI methods we have discussed can be applied to these data to create a wide variety of applications that may add value to primary care patients and providers. It is not guaranteed that AI will transform primary care or any other areas of medicine. Researchers studying this area have emphasized the importance of involving primary care patients, providers and health administrators in the process of developing useful AI applications that address the needs of end users. It is also important to evaluate AI tools to be certain that they actually improve patient care.

There are some limitations to AI that raise important ethical questions. AI algorithms are only as good as the datasets they are trained on. Datasets that poorly represent certain groups may result in AI algorithms that make less accurate predictions for members of those groups, leading to bias. When used in healthcare decision-making, biased predictions may worsen existing social inequalities in health. Issues like consent to data use are also important to consider when applying AI in healthcare settings.

1. **Pre-session questionnaire**

Please return to the online module to complete the questionnaire at least two hours before your first session.

**References**

1. Buchanan BG. *A (Very) Brief History of Artificial Intelligence*. Vol 26.; 2005. doi:10.1609/AIMAG.V26I4.1848

2. Hinton G. Deep Learning — a technology with the potential to transform health care. *JAMA*. 2018;320(11):1101-1102. doi:10.1038/s41551

3. Naylor CD. On the prospects for a (deep) learning health care system. *JAMA*. 2018;320(11):1099-1100. doi:10.1001/jama

4. Lecun Y, Bengio Y, Hinton G. Deep learning. *Nature*. 2015;521(7553):436-444. doi:10.1038/nature14539

5. Topol EJ. High-performance medicine: the convergence of human and artificial intelligence. *Nat Med*. 2019;25(1):44-56. doi:10.1038/s41591-018-0300-7

6. Starfield B, Shi L, Macinko J. Contribution of primary care to health systems and health. *Milbank Q*. 2005;83(3):457-502. doi:10.1111/j.1468-0009.2005.00409.x

7. Wicklund E. Apple Unveils mHealth Integration With EMR Data Through Health App. mHealth Intelligence. Published 2018. Accessed April 21, 2021. https://mhealthintelligence.com/news/apple-unveils-mhealth-integration-with-emr-data-through-health-app

8. Miotto R, Li L, Kidd BA, Dudley JT. Deep Patient: An Unsupervised Representation to Predict the Future of Patients from the Electronic Health Records. *Sci Rep*. 2016;6(1):1-10. doi:10.1038/srep26094

9. Razavian N, Marcus J, Sontag D. *Multi-Task Prediction of Disease Onsets from Longitudinal Lab Tests*. Accessed April 21, 2021. https://github.

10. Shameer K, Johnson KW, Yahi A, et al. Predictive modeling of hospital readmission rates using electronic medical record-wide machine learning: A case-study using Mount Sinai heart failure cohort. In: *Pacific Symposium on Biocomputing*. World Scientific Publishing Co. Pte Ltd; 2017:276-287. doi:10.1142/9789813207813_0027

11. Abràmoff MD, Lavin PT, Birch M, Shah N, Folk JC. Pivotal trial of an autonomous AI-based diagnostic system for detection of diabetic retinopathy in primary care offices. *npj Digit Med*. 2018;1(1). doi:10.1038/s41746-018-0040-6

12. Yang Z, Huang Y, Jiang Y, Sun Y, Zhang YJ, Luo P. Clinical Assistant Diagnosis for Electronic Medical Record Based on Convolutional Neural Network. *Sci Rep*. 2018;8(1):6329. doi:10.1038/s41598-018-24389-w

13. Chekroud AM, Zotti RJ, Shehzad Z, et al. Cross-trial prediction of treatment outcome in depression: A machine learning approach. *The Lancet Psychiatry*. 2016;3(3):243-250. doi:10.1016/S2215-0366(15)00471-X

14. Razzaki S, Baker A, Perov Y, et al. A comparative study of artificial intelligence and human doctors for the purpose of triage and diagnosis. Published online 2018.

15. Lin SY, Mahoney MR, Sinsky CA. Ten Ways Artificial Intelligence Will Transform Primary Care. *J Gen Intern Med*. 2019;34(8):1626-1630. doi:10.1007/s11606-019-05035-1

16. Liaw W, Kakadiaris IA. Artificial intelligence and family medicine: Better together. *Fam Med*. 2020;52(1):8-10. doi:10.22454/FamMed.2020.881454

17. Kueper JK, Terry AL, Zwarenstein M, Lizotte DJ. Artificial Intelligence and Primary Care Research: A Scoping Review. *Ann Fam Med*. 2020;18(3):250-258. doi:10.1370/afm.2518

18. McCradden MD, Sarker T, Paprica PA. Conditionally positive: A qualitative study of public perceptions about using health data for artificial intelligence research. *BMJ Open*. 2020;10(10):39798. doi:10.1136/bmjopen-2020-039798

19. Keane PA, Topol EJ. With an eye to AI and autonomous diagnosis. *npj Digit Med*. 2018;1(1):10-12. doi:10.1038/s41746-018-0048-y

20. Wolff J, Pauling J, Keck A, Baumbach J. The economic impact of artificial intelligence in health care: Systematic review. *J Med Internet Res*. 2020;22(2):e16866. doi:10.2196/16866

21. Martin D, Miller AP, Quesnel-Vallée A, Caron NR, Vissandjée B, Marchildon GP. Canada’s universal health-care system: achieving its potential. *Lancet*. 2018;391(10131):1718-1735. doi:10.1016/S0140-6736(18)30181-8

22. Statistics Canada. Primary health care providers, 2019. Published October 22, 2020. Accessed April 4, 2021. https://www150.statcan.gc.ca/n1/pub/82-625-x/2020001/article/00004-eng.htm

23. Canadian Medical Association. *CMA Workforce Survey 2019: Electronic Records and Tools*.; 2019. https://surveys.cma.ca/en/list?p=1&ps=20&sort=title_sort asc&topic_facet=Electronic records and tools&year_facet=2019

24. McCoy ML, Scully PL. Deliberative Dialogue to Expand Civic Engagement: What Kind of Talk Does Democracy Need? *Natl Civ Rev*. 2002;91(2):117-135.

25. Mulvale G, Chodos H, Bartram M, MacKinnon MP, Abud M. Engaging civil society through deliberative dialogue to create the first Mental Health Strategy for Canada: Changing Directions, Changing Lives. *Soc Sci Med*. 2014;123:262-268. doi:10.1016/j.socscimed.2014.07.029

26. Ridde V, Dagenais C. What we have learnt (so far) about deliberative dialogue for evidence-based policymaking in West Africa. *BMJ Glob Heal*. 2017;2(4):e000432. doi:10.1136/bmjgh-2017-000432

27. Lavis JN, Boyko J, Oxman AD, Lewin S, Fretheim A. SUPPORT Tools for evidence-informed health Policymaking (STP) 14: Organising and using policy dialogues to support evidence-informed policymaking. *Heal Res Policy Syst*. 2009;7(S14). doi:10.1186/1478-4505-7-S1-S14

28. Boyko JA, Lavis JN, Abelson J, Dobbins M, Carter N. Deliberative dialogues as a mechanism for knowledge translation and exchange in health systems decision-making. *Soc Sci Med*. 2012;75(11):1938-1945. doi:10.1016/j.socscimed.2012.06.016

29. Patton M. Purposeful Sampling. In: Laughton C, Axelsen D, Peterson K, eds. *Qualitative Research & Evaluation Methods*. 3rd ed. Sage Publications Ltd.; 2001:230-242.

30. Sittig DF, Singh H. A new sociotechnical model for studying health information technology in complex adaptive healthcare systems. *Qual Saf Heal Care*. 2010;19(1):68-74. doi:10.1136/qshc.2010.042085

31. Sittig DF, Singh H. A new sociotechnical model for studying health information technology in complex adaptive healthcare systems. *Qual Saf Heal Care*. 2010;19:68-74. doi:10.1136/qshc.2010.042085

32. García JF, Hieronimus S, Spatharou A, Beck J-P, Jenkins J. Transforming healthcare with AI The impact on the workforce. *McKinsey Co*. 2020;(March):1-131.

33. Thorne S, SR K, J M-E. Interpretive description: a noncategorical qualitative alternative for developing nursing knowledge. *Res Nurs Heal*. 1997;20(2):169-177.

34. Thorne S, Kirkham S, O’Flynn-Magee K. The Analytic Challenge in Interpretive Description. *Int J Qual Methods*. 2004;3(1). http://www.ualberta.ca/~iiqm/backissues/3_1/ pdf/thorneetal.pdf

35. Plamondon KM, Bottorff JL, Cole DC. Analyzing Data Generated Through Deliberative Dialogue. *Qual Health Res*. 2015;25(11):1529-1539. doi:10.1177/1049732315581603

36. Sangaramoorthy T, Kroeger KA. *Rapid Ethnographic Assessments: A Practical Approach and Toolkit for Collaborative Community Research*. Routledge; 2020.

37. Liyanage H, Liaw ST, Jonnagaddala J, et al. Artificial Intelligence in Primary Health Care: Perceptions, Issues, and Challenges. In: *Yearbook of Medical Informatics*. Vol 28. NLM (Medline); 2019:41-46. doi:10.1055/s-0039-1677901

38. Liaw W, Kakadiaris IA. Artificial Intelligence and Family Medicine: Better Together. *Fam Med*. 2020;52(1):8-10. doi:10.22454/FamMed.2020.881454

39. McCurdie T, Taneva S, Casselman M, et al. mHealth consumer apps: the case for user-centered design. *Biomed Instrum Technol*. 2012;Suppl:49-56. doi:10.2345/0899-8205-46.s2.49

40. Sanders EB-N, Stappers PJ. Co-creation and the new landscapes of design. *CoDesign*. 2008;4(1):5-18. doi:10.1080/15710880701875068

41. Blease C, Bernstein MH, Gaab J, et al. Computerization and the future of primary care: A survey of general practitioners in the UK. Kamolz L-P, ed. *PLoS One*. 2018;13(12):e0207418. doi:10.1371/journal.pone.0207418

42. Blease C, Kaptchuk TJ, Bernstein MH, Mandl KD, Halamka JD, DesRoches CM. Artificial Intelligence and the Future of Primary Care: Exploratory Qualitative Study of UK General Practitioners’ Views. *J Med Internet Res*. 2019;21(3):e12802. doi:10.2196/12802

43. Oh S, Kim JH, Choi SW, Lee HJ, Hong J, Kwon SH. Physician confidence in artificial intelligence: An online mobile survey. *J Med Internet Res*. 2019;21(3):e12422. doi:10.2196/12422

44. Giunti G, Guisado-Fernandez E, Belani H, Lacalle-Remigio JR. Mapping the access of future doctors to health information technologies training in the european union: Cross-sectional descriptive study. *J Med Internet Res*. 2019;21(8):e14086. doi:10.2196/14086

45. Paranjape K, Schinkel M, Rishi ;, Panday N, Car J, Nanayakkara P. Introducing Artificial Intelligence Training in Medical Education. *JMIR Med Educ*. 2019;5(2):16048. doi:10.2196/16048

46. Kolachalama VB, Garg PS. Machine learning and medical education. *npj Digit Med*. 2018;1(1):1-3. doi:10.1038/s41746-018-0061-1

47. Rampton V, Mittelman M, Goldhahn J. Implications of artificial intelligence for medical education. *Lancet Digit Heal*. 2020;2(3):e111-e112. doi:10.1016/S2589-7500(20)30023-6

48. Harish V, Aissiou A, Macmillan K, Mcleod G, Ngo R, Yau O. *Preparing Medical Students for the Impact of Artificial Intelligence on Healthcare Introduction*.; 2019.

49. Task Force 1. Report of the Task Force on Patient Expectations, Core Values, Reintegration, and the New Model of Family Medicine. *Ann Fam Med*. 2004;2(suppl_1):S33-S50. doi:10.1370/afm.134

50. OECD. Adult education level (indicator). doi:10.1787/36bce3fe-en

51. OECD. Poverty rate (indicator). doi:OECD (2021), Poverty rate (indicator). doi: 10.1787/0fe1315d-en (Accessed on 29 April 2021)

52. Walter M, Lovett R, Maher B, et al. Indigenous data sovereignty in the era of Big Data and Open Data. *Aust J Soc Issues*. Published online 2020.

53. Boyko JA, Lavis JN, Dobbins M. Deliberative dialogues as a strategy for system-level knowledge translation and exchange. *Healthc Policy*. 2014;9(4):122-131. doi:10.12927/hcpol.2014.23808

54. Government of Canada. About primary health care. Published 2012. Accessed September 4, 2020. https://www.canada.ca/en/health-canada/services/primary-health-care/about-primary-health-care.html

55. Upshur R. *Artificial Intelligence, Machine Learning and The Potential Impacts on the Practice of Family Medicine: A Briefing Document*.; 2019.

56. Hutson M. AI Glossary: Artificial intelligence, in so many words. *Science (80- )*. 2017;357(6346). doi:10.1126/science.357.6346.19

57. Shaw J, Rudzicz F, Jamieson T, Goldfarb A. Artificial Intelligence and the Implementation Challenge. *J Med Internet Res*. 2019;21(7):e13659. doi:10.2196/13659

58. Buchanan BG. A (Very) Brief History of Artificial Intelligence. *AI Mag*. 2005;26(4):53. doi:10.1609/aimag.v26i4.1848

59. Davidson L. Narrow vs. General AI: What’s Next for Artificial Intelligence? Springboard. Published 2019. Accessed September 4, 2020. https://www.springboard.com/blog/narrow-vs-general-ai/#:~:text=Narrow AI (ANI) is defined,and advances in that spectrum.”

60. Beam AL, Kohane IS. Big Data and Machine Learning in Health Care. *J Am Med Assoc*. 2018;319(12):1317-1318. doi:10.1001/jama.2017.18391

61. Teng J, Burgess M, McGrail K, Bentley C, O’Doherty K. *Research Data Use in a Digital Society: A Deliberative Public Engagement*.; 2019.

62. Esteva A, Kuprel B, Novoa RA, et al. Dermatologist-level classification of skin cancer with deep neural networks. *Nature*. 2017;542(7639):115-118. doi:10.1038/nature21056

63. Topol EJ. High-performance medicine: the convergence of human and artificial intelligence. *Nat Med*. 2019;25(1):44-56. doi:10.1038/s41591-018-0300-7

64. Rajkomar A, Oren E, Chen K, et al. Scalable and accurate deep learning for electronic health records. *npj Digit Med*. 2018;(January):1-10. doi:10.1038/s41746-018-0029-1

65. Chang F, Gupta N. Progress in electronic medical record adoption in Canada. *Can Fam Physician*. 2015;61(December):1076-1084.

66. Kueper JK, Terry AL, Zwarenstein M, Lizotte DJ. Artificial intelligence and primary care research: A scoping review. *Ann Fam Med*. 2020;18(3):250-258. doi:10.1370/afm.2518

67. Hill RG, Sears LM, Melanson SW. 4000 Clicks: A productivity analysis of electronic medical records in a community hospital ED. *Am J Emerg Med*. 2013;31(11):1591-1594. doi:10.1016/j.ajem.2013.06.028

68. Wachter R, Goldsmith J. To Combat Physician Burnout and Improve Care, Fix the Electronic Health Record. *Harvard Bus Rev Digit Artic*. Published online 2018:2-5. doi:10.1056/NEJMp1102099

69. Birch M, Folk JC, Shah N, Lavin PT, Abràmoff MD. Pivotal trial of an autonomous AI-based diagnostic system for detection of diabetic retinopathy in primary care offices. *npj Digit Med*. 2018;1(1). doi:10.1038/s41746-018-0040-6

70. Marcus JL, Hurley LB, Krakower DS, Alexeeff S, Silverberg MJ, Volk JE. Use of electronic health record data and machine learning to identify candidates for HIV pre-exposure prophylaxis: a modelling study. *lancet HIV*. 2019;6(10):e688-e695. doi:10.1016/S2352-3018(19)30137-7

71. Saykara. AI Assistant for Physicians. Published 2020. Accessed September 5, 2020. https://www.saykara.com/

72. Wiens J, Saria S, Sendak M, et al. Do no harm: a roadmap for responsible machine learning for health care. *Nat Med*. 2019;25(9):1337-1340. doi:10.1038/s41591-019-0548-6

73. Obermeyer Z, Powers B, Vogeli C, Mullainathan S. Dissecting racial bias in an algorithm used to manage the health of populations. *Science (80- )*. 2019;366:447-453.

**Glossary**

**Administrative data**Data collected in the course of providing and/or paying for services (*e.g.* hospital admissions, physician visits)

**Algorithm** A set of step-by-step instructions for solving a problem. A computer algorithm can be simple (e.g. If it’s Saturday at 9 AM, send a reminder) or complex (e.g. Identify pedestrians).

**Artificial intelligence**A broad field of science that is concerned with getting computers to do tasks that would normally require human intelligence; a*-general purpose prediction technology* that estimates missing information from prior information.

**Bias** A preference for one thing, idea, person or group compared to another, usually in a way that is considered unfair

**Clinical data** Data collected by a hospital or provider in order to provide appropriate healthcare services

**Computer vision** AI algorithms that interpret digital images or videos

**Dataset** A collection of data gathered using the same criteria for a specific purpose

**De-identifed data**Data where identifiers such as a person’s name and address have been removed to reduce the possibility that the data could be traced back to any individual

**Deep learning**A subgroup of machine learning algorithms that identify hidden patterns in a dataset. These patterns can be used either to classify new data into a predefined category (*i.e.* using supervised learning methods) or group new data by similarity

**Deliberative dialogue** A discussion among people involved in or affected by future decisions about a high-priority issue. A dialogue aims to bring stakeholders together to understand a topic in greater depth and share different views.

**Electronic medical record**Digital records kept by hospitals or individual providers that contain clinical data

**Explainable AI** AI algorithms where the features of a dataset that contribute to a pattern are possible to identify and define

**General AI** A single system that can learn in different situations and then apply broad knowledge to solve any kind of problem. There are currently no actual examples of general AI.

**Genomic data**Data describing DNA sequences

**Linked dataset** Dataset created when two datasets from different sources are combines. Linked datasets contain a broader range of information than either of the original datasets.

**Machine learning** AI methods where a computer program learns from experience over time

**Narrow AI**AI programs that can only do what they were designed to do. "Narrow" AI applications are often better than humans at the tasks they were designed for but cannot develop additional skills without being programmed by humans.

**Natural language processing**AI methods used to interpret and reproduce human communication

**Non-explainable AI**AI algorithms where the features of a dataset that contribute to a pattern are difficult or impossible to identify and define

**Physical data**Data about how your body is functioning from moment-to-moment, such as your heart rate of blood glucose levels; often collected from wearable devices

**Primary care** The services involved in health promotion, illness and injury prevention, and the diagnosis and treatment of illness and injury; usually the point of first contact with a healthcare provider when you are sick or injured

**Risk stratification** Organizing people into risk levels that can be used to guide decisions. Risk stratification can be developed by

**Supervised learning**A type of machine learning where an algorithm learns from labelled examples or positive and negative cases. Supervised learning is used to assign data into predetermined categories.

**Unsupervised learning**A type of machine learning where an algorithm learns without examples. Unsupervised learning is used to group data by similarities.
