# Supplementary material for: Implementing artificial intelligence in Canadian primary care: Barriers and strategies identified through a national deliberative dialogue
Source: PLoS One. 2023 Feb 27;18(2):e0281733. doi: 10.1371/journal.pone.0281733 (PMC9970060; doi:10.1371/journal.pone.0281733)
Supplement: Attachment — Submitted filename: AI for primary care - PLOS ONE - response to reviewers.docx [file pone.0281733.s005.docx]

**Andrew D. Pinto** MD CCFP FRCPC MSc

CIHR Applied Public Health Research Chair

Director, Upstream Lab, MAP / Centre for Urban Health Solutions, Li Ka Shing Knowledge Institute, Unity Health Toronto

Physician, Department of Family & Comm. Medicine, St. Michael’s Hospital

Associate Professor, Department of Family & Comm. Medicine, Faculty of Medicine, University of Toronto

Associate Professor (Status), Institute for Health Policy, Management and Evaluation

Associate Professor (Status), Dalla Lana School of Public Health, University of Toronto

Associate Director of Clinical Research, University of Toronto Practice-Based Research Network (UTOPIAN)

November 7, 2022

Re: **Manuscript PONE-D-22-20397**

Dear PLoS ONE Editorial Team,

We appreciate the time taken by our reviewers and the editorial team to review our manuscript entitled, *Implementing artificial intelligence in Canadian primary care: barriers and strategies identified through a national deliberative dialogue*. We appreciate the opportunity to resubmit this paper, and we have addressed each concern raised.

We look forward to hearing from you about this manuscript.

Sincerely,

Andrew D. Pinto MD CCFP FRCPC MSc

**Reviewer #1:**

| 1. There needs to be more examples of existing uses of AI in the introduction to put the current work in better context. In relation to this, any examples of AI application in primary care that were discussed in the dialogues should be expanded on if it would help explain the existing comments and quotes by participants. | - Examples added: “Examples of risk prediction AI tools applicable to the primary care space include machine learning algorithms that identify primary care patients at risk for cancer (3), predict which patients may experience post-partum depression (4,5), and estimate those who may be at risk of multiple chronic diseases including diabetes, osteoarthritis, and hypertension (6,7). Although a flurry of research is focused on the development of AI tools, implementation into practice remains one of the most challenging barriers to overcome.” - Applications discussed in the dialogue were theoretical cases to provide a basis for discussion. - Also added more literature review on AI implementation challenges in health care:   “A recent review by Lovejoy et al. explores these challenges to health care as a whole by breaking down the AI innovation process into three stages – invention, development, and implementation. Specifically with respect to the implementation stage, they highlight three main areas of focus (generalizability, regulation, and deployment). Generalizability refers to the need for diversity within the training set, regulation refers to the need for ongoing monitoring of an algorithm or tool throughout its lifetime to ensure continued safety, and deployment refers to the need to minimize disruption to workflow and account for interoperability between health care technologies. Although this review is not specific to the primary care space, it provides insight into some of the barriers to AI implementation that exist within the health care space. Another review by Singh et al. outlines the barriers in relation to the perspective of health care organizations, health care providers, and patients. Interestingly, from the health care organization perspective, they discuss the role of “company culture” as important when considering the use of embracing AI in their day-to-day practice. The provider perspective outlined concerns for the consideration of racial, ethnic, gender, and other sociodemographic characteristics within the algorithm. Finally, the patients perspective was based on trust, and what physician-patient confidentiality would look like when AI is used in their care. Although these reviews are not specific to the primary care space, they provide insight into some of the barriers to AI implementation that exist within the health care space.” |
| --- | --- |
| 2. I note with interest the sections mentioning the limitation of processing longitudinal clinical data for AI use, and think this is worth expanding on as a significant practical difficulty. | - Just to clarify, the introduction notes that longitudinal clinical data is one of the reasons that is unique to primary care data, but not necessarily a limitation. |
| 3. It would benefit your international audience to explain more about the health system in Canada as well as the terminology you have used to describe it e.g. what is a team-based care model? | - Re-wrote the “Setting” section to be more comprehensive of the models used in Canada, and to explain that healthcare is provincially/territorially managed:   “This study was conducted virtually within Canada, a high-income country with provincially and territorial-based public health insurance that covers all necessary hospital and physician services (5). Both within and across provinces and territories of Canada, primary health care is delivered through different models. Largely, it is funded following a fee-for-service (FFS) model, but has adopted new funding models such as the enhanced fee-for-service model and capitation model. In Ontario, the enhanced FFS model is similar to the traditional FFS model, except that it includes additional targeted fee increases, extended hours, performance-based initiatives, and patient enrollment. In contrast, the capitation model reimburses primary care physicians for each person attributed to them, based on age and sex adjusted factors (6). More recently, Ontario has been forming Ontario Health Teams including family physicians, nurse practitioners, social workers, dietitians, and other health care professionals to increase interdisciplinary care for a given community (7).” |
| 4. What is the quadruple aim and support equity? | - Supporting equity put before quadruple aim so it did not sound like it was all one thing. - Added definition within text to clarify: “optimizing health system performance through improved patient experience, improved clinical experience, better outcomes, and lower costs” |
| 5. Please provide the participant information sheets handed out before the dialogues as supplemental data | - AI learning module will be attached when revisions are re-uploaded. It is named “Supplemental Material – Participant AI Learning Module” |
| 6. Please list which advertisement platforms were used and acknowledge any patient groups that were used. | - Added this to the manuscript under Participants section:   “Specifically, patients were recruited using Kijiji and social media channels across 10 provinces as well as patient advisor distribution lists (patient advisory network in British Columbia, patient-family advisory council of downtown Toronto hospitals). Primary care physicians were recruited through social media channels, primary care research network news channels, and through email invitations from AP to practice colleagues and those in his network interested in digital health and health informatics.” |
| 7. More details on sampling method would be beneficial | - Added more detail for sampling method: “This recruitment method is outlined further by Patton et al (21), and was chosen in order to optimize variation in age, gender, race and ethnicity, education level, income, province of residence for patients and gender, race and ethnicity, provider type, country of health professions education, years in practice, practice size, and province of practice for primary care providers.” |
| 8. Line 227 "publics" should be "populations" | - Switched from “publics” to “populations” |
| 9. It would be useful to write provide a further description of sittig and sing's sociotechnological model which was used as a framework for analysis. In particular, why was it chosen in lieu of other prevailing models such as the unified theory of acceptance and use of technology (UTAUT), and technology acceptance model (TAM)? | - Revised intro of model: “Sittig and Singh’s sociotechnological model for studying health information technology in complex adaptive healthcare systems demonstrates the complex challenges involved in the design, development, implementation, use, and evaluation of technology in health care settings. It was designed to address the both the sociological and technological challenges that are present within health care settings that are high-pressured, fast-paced and distributed.” - Added explanation: “This model was used as a comparison of our four themes and was chosen over other models such as the Technology Acceptance Model (TAM) and the Unified Theory of Acceptance and Use of Technology (UTAUT) for its applicability to health care. The TAM and UTAUT aim to understand why users accept or reject a given technology and how acceptance can be improved, but recent reviews show that they have failed to predict acceptance of technologies in health care (23). This may be because of health care’s unique cultural, social, and organizational factors that influence technology adoption. Therefore, Sittig and Singh’s model remained the most appropriate choice for our comparison due to its specificity to health information technology.” |
| 10. If there are any coding tables available to visualise how the final themes were reached from the primary codes, these would be useful to put into supplementary data so that the iterative process can be demonstrated. | - Coding table attached to re-submission |

**Reviewer #2:**

| This is a well written article on an important topic. The main barrier to publication, in my view, is the weakness of the literature review. There are other papers which have explored barriers to implementation, for example: https://doi.org/10.7861/fhj.2021-0128 and https://www.ncbi.nlm.nih.gov/pmc/articles/PMC7443115/ This paper is good opportunity to highlight challenges raised by existing literature with empirical evidence. The Introduction section is very concise and superficial at the moment. Please consider expanding it with more detailed overview of existing literature. The authors write: "There is a paucity of research that focuses specifically on AI implementation challenges and the strategies to overcome them within the primary care space." but they do not really mention any of the existing research in the field. There are only four references in the introduction and all of these are related to generic AI papers. I think that with an expanded literature review in the discussion and an enhanced introduction, this paper would be a good addition to the body of literature on AI implementation science. | - Added findings by one of the reccomended papers by Reviewer #2: “A recent review by Lovejoy et al. explores these challenges to health care as a whole by breaking down the AI innovation process into three stages – invention, development, and implementation. Specifically with respect to the implementation stage, they highlight three main areas of focus (generalizability, regulation, and deployment). Generalizability refers to the need for diversity within the training set, regulation refers to the need for ongoing monitoring of an algorithm or tool throughout its lifetime to ensure continued safety, and deployment refers to the need to minimize disruption to workflow and account for interoperability between health care technologies. Although this review is not specific to the primary care space, it provides insight into some of the barriers to AI implementation that exist within the health care space.” - Also from the second recommended manuscript from reviewer #2: “Another review by Singh et al. outlines the barriers in relation to the perspective of health care organizations, health care providers, and patients. Interestingly, from the health care organization perspective, they discuss the role of “company culture” as important when considering the use of embracing AI in their day-to-day practice. The provider perspective outlined concerns for the consideration of racial, ethnic, gender, and other sociodemographic characteristics within the algorithm. Finally, the patients perspective was based on trust, and what physician-patient confidentiality would look like when AI is used in their care. Although these reviews are not specific to the primary care space, they provide insight into some of the barriers to AI implementation that exist within the health care space.” |
| --- | --- |
