# Supplementary material for: Implementing artificial intelligence in Canadian primary care: Barriers and strategies identified through a national deliberative dialogue
Source: PLoS One. 2023 Feb 27;18(2):e0281733. doi: 10.1371/journal.pone.0281733 (PMC9970060; doi:10.1371/journal.pone.0281733)
Supplement: Attachment — Submitted filename: Response-to-Reviewers.docx [file pone.0281733.s006.docx]

**Andrew D. Pinto** MD CCFP FRCPC MSc

CIHR Applied Public Health Research Chair

Director, Upstream Lab, MAP / Centre for Urban Health Solutions, Li Ka Shing Knowledge Institute, Unity Health Toronto

Physician, Department of Family & Comm. Medicine, St. Michael’s Hospital

Associate Professor, Department of Family & Comm. Medicine, Faculty of Medicine, University of Toronto

Associate Professor (Status), Institute for Health Policy, Management and Evaluation

Associate Professor (Status), Dalla Lana School of Public Health, University of Toronto

Associate Director of Clinical Research, University of Toronto Practice-Based Research Network (UTOPIAN)

January 5^th^, 2023

Re: **Manuscript PONE-D-22-20397**

Dear PLoS ONE Editorial Team,

We appreciate the time taken by our reviewers and the editorial team to review our manuscript entitled, *Implementing artificial intelligence in Canadian primary care: barriers and strategies identified through a national deliberative dialogue*. We appreciate the opportunity to resubmit this paper, and we have addressed each concern raised.

We look forward to hearing from you about this manuscript.

Sincerely,

Andrew D. Pinto MD CCFP FRCPC MSc

**Reviewer #2:**

| My original review focussed on two deficiencies: the brevity of the Introduction and of the Discussion. The authors have adequately expanded the Introduction and I hope they agree that this has strengthened the manuscript. However, the lack of comparison to existing literature in the Discussion remains a weakness of the manuscript. The Discussion section of the manuscript remains sparse and there is only one other relevant paper discussed: "Sittig and Singh’s sociotechnological model for studying health information technology". It would be advisable to research previous studies using surveys and or other similar methodologies to identify barriers to limitations. | - Thank you for the guidance on the introduction. We agree that the changes to the introduction have improved the paper greatly. - Some changes to the discussion were made in the last round of edits (introducing the TAM and UTAUT models and why Sittig & Singh was selected over them) - Changes to the discussion during this round of edits includes a more in-depth comparison to the Sittig & Singh model:   “The system and data readiness theme does not align strongly with any one given dimension but correlates loosely with both the “hardware and software computing infrastructure” and “clinical content” dimensions. The former covers the technological perspective, including equipment and software, which could be extended to cover the contents of our theme including interoperability and digitization of medical records, while the latter addresses personal health information, data quality, and data structure.  The potential for bias and inequity theme is also not explicitly present in Sittig and Singh’s eight dimensions, possibly because AI has the potential to present biases in ways that technology was less capable of before. Furthermore, trust, the overarching foundation to all of our themes is also not present in the eight dimensions but was very important to the participants during the dialogue sessions.  Overall, Sittig and Singh’s sociotechnological framework is advantageous to consider both the social and technical perspectives of technology implementation projects, but based on our deliberative dialogue series, we believe that new dimensions (e.g., trust, bias, and equity) would be required to translate this model to be applicable to AI implementation projects in the primary care space now and in the future.”   - Further research was completed to compare our finding to two other reviews on barriers to implementation:   “As mentioned in the introduction, the recent review conducted by Lovejoy et al. divides the AI innovation process into three stages (invention, development, and implementation) and outlines key considerations for innovators during each phase (13). The considerations of the third stage, implementation, include generalizability, regulation, and deployment. Generalizability refers to the need for diversity within the training set, regulation refers to the need for ongoing monitoring of an algorithm or tool throughout its lifetime to ensure continued safety, and deployment refers to the need to minimize disruption to workflow and account for interoperability between healthcare technologies (13). When comparing the four themes uncovered in our study to the implementation stage of this review, it is evident that our deliberative dialogue results greatly support those of Lovejoy et al. For example, within our ‘potential for bias and inequity’ and ‘regulation of AI and big data’ themes, we heard multiple times in our sessions that diversity within training sets and continuous monitoring of a given tool are both vital to ensure bias and safety are maintained over time. This is very similar to Lovejoy et al.’s generalizability and regulation sections as they state, “The training data must be at least as diverse as the population that the algorithm intends to serve” and “Good performance at the time of deployment does not guarantee that the model will continue to perform well. This introduces the need to regulate throughout the lifetime of an algorithm, and the need to continually demonstrate safe and effective practices” (13). Therefore, our ‘potential for bias and inequity’ theme is similar to Lovejoy et al.’s concept of generalizability, and our ‘regulation of AI and big data’ compliments the regulation section explained in their review.  The review by Singh et al. outlines AI implementation barriers in relation to the perspective of healthcare organizations, healthcare providers, and patients. These three perspectives are similar to those included in our study, except that instead of healthcare organizations we heard from health system leaders. From the review’s healthcare organization, they discussed the role of “company culture” as important when considering the use of embracing AI in their day-to-day practice – a notion that did not come up explicitly in our deliberative dialogue sessions. The provider perspective outlined concerns for the consideration of racial, ethnic, gender, and other sociodemographic characteristics within the algorithm. This was similar to what was heard in our ‘Potential for bias and inequity’ theme, except that we heard the concern from all three types of stakeholders and not just from the providers alone. The perspective of patients was centered on trust, including the concern for physician-patient confidentiality if AI were to be used in their care (14). This compliments our finding of the importance of trust, however, in our sessions it was woven into all four themes and from all perspectives (patients, providers and health system leaders).”   - See below for additional changes made to the discussion section. |
| --- | --- |

**Reviewer #3:**

| The paper is sufficiently innovative but needs some improvement as follows to be ready for publication in the journal:  1. The comparison with other suggested or conventional methods and their discussion is not well-highlighted in the introduction. It can helps to demonstrate the strength of the work and its weakness practically. | - Introduced the common tech implementation models (TAM and UTUAT). Also introduced the Sittig and Singh model in the introduction. These three models are referred to in the discussion so we agree, it is important to introduce them first in the introduction.   “To identify and ease these barriers, researchers have developed models related to technology implementation including the ‘Technology Acceptance Model’ (TAM) and the ‘Unified Theory of Acceptance and the Use of Technology’ (UTAUT) model (10,11). These are well studied models that explore the reasons that users accept or reject a given technology and how acceptance can be improved, but unfortunately are not specific to primary healthcare or even to the healthcare at all. Another model, Sittig and Singh’s sociotechnological model for studying health information technology includes eight dimensions (hardware and software computing infrastructure, clinical content, human-computer interface, people, workflow and communication, internal organizational features, external rules and regulations, and measurement and monitoring (12). Sittig and Singh’s model focuses specifically on implementation of technology in complex adaptive healthcare systems and will be used as a basis of comparison for the findings of this study.”   - Additionally, the mention of the two reviews in the introduction (Lovejoy et al. and Singh et al.) were better highlighted and explained in relation to this paper.   “Other researchers including Lovejoy et al. and Singh et al. have conducted reviews to explore the challenges of implementing AI in healthcare settings. The recent review by Lovejoy et al. explores the challenges of implementing AI to healthcare by breaking down the AI innovation process into three stages – invention, development, and implementation. Specifically with respect to the implementation stage, they highlight three main barriers of focus (generalizability, regulation, and deployment). Generalizability refers to the need for diversity within the training set, regulation refers to the need for ongoing monitoring of an algorithm or tool throughout its lifetime to ensure continued safety, and deployment refers to the need to minimize disruption to workflow and account for interoperability between healthcare technologies (13). Although this review is not specific to the primary care space, it provides insights into barriers that are commonly encountered in the healthcare AI space, all of which will help inform our study. Another review by Singh et al. outlines the barriers in relation to the perspective of healthcare organizations, healthcare providers, and patients. Interestingly, from the healthcare organization perspective, they discuss the role of “company culture” as important when considering the use of embracing AI in their day-to-day practice. The provider perspective outlined concerns for the consideration of racial, ethnic, gender, and other sociodemographic characteristics within the algorithm. The perspective of patients was centered on trust, including the concern for physician-patient confidentiality if AI were to be used in their care (14). This review provides vital insight from various stakeholder perspectives and emphasizes the importance of doing so.” |
| --- | --- |
| 2. Neural network and fuzzy networks are very efficient intelligent methods. How can the offered method be improved by using fuzzy systems and neural networks? It is strongly recommended to include the following paper in the introduction refer and explain. An interval type-3 fuzzy system and a new online fractional-order learning algorithm: theory and practice. | - We appreciate this recommendation. We have given it careful consideration, and believe the suggestion citation and related recommendation for revision are beyond the scope of the work presented in this manuscript. |
| 3. "Only five health system leaders were included in the study and no self-identifying Indigenous people". According to the mentioned limitation, what is the suitable practical solution applicable for the future works improvement? | - Added a practical solution for future work:   “To further improve this study and its transferability, future work should focus on the perspectives of those that self-identify as indigenous as well as individuals form Canada’s territories. This would enable more diverse perspectives and would be vital before any AI implementation projects are considered.” |
| 4. Is this level of study of the mentioned community effective for practical implementations? Please explain? | - Expanded on this as follows:   “This level of study (48 participants from 8 Canadian provinces including 22 patients, 21 primary care providers, and 5 health system leaders) provides an excellent starting point for the barriers and strategies to use when implementing AI in primary care settings, but a study with larger reach would be recommended before practical AI applications become commonplace in primary care. To increase reach and gain further insight, a deliberative dialogue would not be practical but instead a survey could gain the perspectives of more of the population. This in addition to a focused dialogue with individuals from Canada’s territories and those who self-identify as indigenous would be vital before practical AI solutions are implemented widely in primary care.” |
